# A Digital Toolkit for Weight Loss Maintenance in European Adults (NoHoW): 2×2 Factorial Randomized Controlled Trial

**DOI:** 10.2196/69634

**Published:** 2026-05-20

**Authors:** R James Stubbs, Cristiana Duarte, Clarissa Dakin, António L Palmeira, Falko F Sniehotta, Graham W Horgan, Sofus C Larsen, Marta M Marques, Jorge Encantado, Elizabeth H Evans, Jake Turicchi, Ruari O'Driscoll, Sarah E Scott, Beth Pearson, Lauren Ramsey, Marie-Louise Mikkelsen, Inês Santos, Marcela Matos, Pedro J Teixeira, Berit L Heitmann

**Affiliations:** 1 School of Psychology, Faculty of Medicine and Health, University of Leeds, University Rd, Woodhouse, Leeds, LS2 9JT, United Kingdom, 44 07955878763; 2 School of Education, Language and Psychology, York St John University, , York, United Kingdom; 3 Centro de Investigação em Desporto Educação Física, Exercício e Saúde (CIDEFES), Universidade Lusófona and Centro de Investigação, Formação, Inovação e Intervenção em Desporto (CIFI2D), Universidade do Porto, Porto, Portugal; 4 Division of Public Health, Social and Preventive Medicine, Centre for Preventive Medicine and Digital Health (CPD), Medical Faculty Mannheim, University of Heidelberg, Mannheim, Germany; 5 Biomathematics and Statistics Scotland, Aberdeen, United Kingdom; 6 Research Unit for Diet and Health at The Parker Institute, Bispebjerg and Frederiksberg Hospital, The Capital Region, Copenhagen, Denmark; 7 NOVA National School of Public Health, Public Health Research Centre, Comprehensive Health Research Center, CHRC, REAL, CCAL, NOVA University Lisbon, Lisbon, Portugal; 8 Interdisciplinary Center for the Study of Human Performance (CIPER), Faculdade de Motricidade Humana, Universidade de Lisboa, Cruz Quebrada, Portugal; 9 Department of Psychology, Faculty of Science, Durham University, Durham, United Kingdom; 10 Faculdade de Medicina, Universidade de Lisboa, Lisboa, Portugal; 11 Instituto de Saúde Ambiental (ISAMB), Faculdade de Medicina, Universidade de Lisboa, Lisboa, Portugal; 12 Center for Research in Neuropsychology and Cognitive and Behavioural Intervention (CINEICC), Universidade de Coimbra, Coimbra, Portugal; 13 Section for General Medicine, The Department of Public Health, University of Copenhagen, Copenhagen, Denmark

**Keywords:** emotion regulation, information and communication technologies, motivation, obesity, self-regulation, weight loss maintenance

## Abstract

**Background:**

Digital approaches to weight management have the potential to produce cost-effective and scalable weight management solutions. Effective behavior change interventions typically promote self-regulation of energy balance behaviors, which may be enhanced by incorporating emotion regulation strategies.

**Objective:**

This study aimed to evaluate the effectiveness of a digital behavior change toolkit for weight loss maintenance in European adults who had achieved ≥5% weight loss in the previous 12 months. We hypothesized that a combined intervention targeting self-regulation or motivation and emotion regulation would be more effective than either component alone, and that each would outperform an active control.

**Methods:**

The Navigating to a Healthier Weight (NoHoW) trial was a 2×2 factorial randomized, single-blind, controlled trial involving 1627 adults who had achieved ≥5% weight loss in the previous 12 months (initial BMI ≥25 kg/m^2^) across 3 European centers (the United Kingdom, Denmark, and Portugal). The trial evaluated a digital toolkit for weight management subsequent to an initial ≥5% weight loss in the prior 12 months. Participants were assigned using adaptive stratified sampling to one of four groups: (1) self-regulation or motivation (n=403), (2) emotion regulation (n=416), (3) combined motivation and emotion (n=408), or (4) active control (generic content, regular self-weighing, and Fitbit use, n=400). The primary outcome was weight change from baseline to 12 months. Prespecified secondary outcomes included cardiometabolic markers. Linear models adjusted for recruitment center, sex, age group, BMI group, and pretrial weight loss. Subgroup analyses were conducted by sex.

**Results:**

At 12 months, 76% (364/1627) of participants remained in the study. In the primary ITT analysis in all participants, none of the intervention arms (motivation, emotion, or combined) differed significantly from the active control for weight change at 12 months. Completer and per-protocol analyses produced similar patterns and did not change the overall interpretation. In the per-protocol sample, men regained 0.14 kg, and women regained 0.54 kg of their pretrial weight loss. Subgroup analyses indicated a small effect of the motivation intervention in men, but this was not clinically meaningful and did not alter the primary null findings. Nearly half of ITT participants regained weight, and no significant intervention effects were observed for cardiometabolic secondary outcomes.

**Conclusions:**

The NoHoW trial was the first large-scale, multicountry 2×2 factorial randomized controlled trial to evaluate a digital-only toolkit based on self-regulation or motivation and emotion regulation techniques for weight loss maintenance. NoHoW found no evidence in the primary ITT analysis that digital interventions targeting self-regulation or emotion regulation improved weight loss maintenance compared with the active control. A small subgroup effect in men should be interpreted cautiously and does not change this conclusion. The trial provides evidence on both the limitations and potential of digital behavior change interventions for long-term weight outcomes. Future digital interventions may benefit from enhanced engagement and tailored content to improve long-term weight outcomes.

## Introduction

### Background

The societal challenges of overweight and obesity affect more than half of the adult population [1]. The health care consequences and associated economic costs are well documented [2,3]. More than 40% of Western adults report making at least 1 weight control attempt per year [4], most of which do not involve evidence-based behavior change approaches [4]. Existing community-based programs support initial weight loss but are subject to high attrition and weight regain, limiting longer-term effectiveness [5-7]. The obesogenic environment and asymmetry of human energy balance regulation facilitate weight gain, while society stigmatizes people experiencing overweight and obesity, leading to stress and negative emotions, which can undermine weight loss attempts [8-10]. The majority of weight loss attempts are followed by some degree of weight regain [11], emphasizing the need to develop more effective weight loss maintenance solutions. Long‑term weight loss maintenance remains a significant challenge, with weight regain highly prevalent. These recurring patterns emphasize the classification of obesity as a chronic, relapsing disease [12,13]. Effective support for weight loss maintenance needs to address sustained changes in energy balance behaviors (diet, physical activity, and weight control) and emotional or psychosocial challenges that may undermine longer-term self-management of those behaviors [14]. Pathways of planned behavior change involving self-management of energy balance behaviors are difficult to implement unless they become practiced and habitual. Dual process models of behavior change suggest that such practice takes time. Existing habits, preferences, urges, desires, and responsiveness to environmental cues often undermine the implementation of new behavior changes [15]. Models of behavior change increasingly include both reflective and reactive elements, which, if not aligned, can deplete psychological resources and undermine maintenance of behavior change [16]. Greaves et al [14] describe longer-term weight management as generating a tension between new (weight management) behaviors and the fulfillment of preexisting psychological needs from existing patterns of behavior. This tension could be managed through self-regulation, renewed motivation, and managing external influences to change habits, finding nonobesogenic approaches to meet psychological needs, and changing self-concept [14]. Some of the factors that undermine longer-term weight loss, such as changes in energy balance physiology affecting energy expenditure, food reward-based processes, or energy intake, may be outside of conscious recognition and control [17]. Aspects of self-regulation and motivation may improve the odds of changing energy balance behaviors, and if those changes become habitual, potentially help prevent weight regain [18-20]. Changing habits can take 2‐5 years [21]. However, reactive processes (emotions, desires, impulses resulting from associative learning, and physiological resistance to weight loss) are powerful forces that can also undermine relatively transient and fragile attempts at changing energy balance behaviors during weight loss.

### Self-Regulation and Motivation

Research identifying and linking specific behavior change approaches to mechanisms of action of behavior change interventions is still a developing field [22,23]. Core features of effective weight loss maintenance interventions include (1) self-monitoring of weight and behavior, (2) goal-setting: agreement of clear weight targets or trigger points for weight control efforts, (3) feedback on behavior and weight, (4) action plans for weight control through dietary and physical activity behaviors, and (5) plans to cope with risk factors for weight regain and relapse prevention (eg, problem-solving) [18-20,24,25]. Many people also experience behavioral lapses and relapses as more pronounced situational or momentary events. Avoiding weight regain requires behavioral strategies, in which relapse coping and weight loss maintenance become learned skills of self-regulation, autonomy, and motivation as part of a longer-term process [12,26-28].

Physical activity and dietary weight loss maintenance interventions based on current behavior change theories characteristically achieve relatively modest effects of ~1.6 kg difference in weight regain compared to unsupported controls over 12 months [18,29,30]. Additional psychological processes, such as emotion regulation, potentially have additional beneficial effects on the behavioral changes that promote weight loss maintenance [14].

### Emotion Regulation

Reactive processes (emotions, desires, habits resulting from associative learning, and physiological states) may have a large impact on behavior change. These relatively rapid, automatic, impulsive (less conscious), and habitual processes (compared to the slow, deliberative processes of motivation and self-regulation) [15,31] may undermine initial self-regulation of energy balance (particularly eating) behaviors in the face of a physiological system that resists longer-term weight loss [32]. Automatic components of self-regulation may also promote longer-term behavior change if they are engaged and developed [33,34]. People with overweight and obesity commonly experience stigma, which enhances psychosocial stress and impacts physical and mental well-being [35-37]. Stigma operates via shame, self-criticism, and unfavorable social comparisons, creating feelings of inferiority and inadequacy in relation to others [38]. The relationship between stress, emotion, and food intake can derail strategies of planned behavior and promote further weight gain [39]. Limited evidence suggests that acceptance, self-compassion, and mindfulness-based approaches could potentially help some people in coping with obesity-related eating behaviors [40,41]. We hypothesized that self-monitoring, self-regulation, and autonomous motivation in weight loss maintenance could be supported favorably by strategies that promote emotion regulation and that may thereby increase coping with stress.

### Digital Weight Loss Maintenance Interventions

Evidence of the effectiveness of long-term digital interventions and strategies to support weight loss maintenance is limited [42]. Digital solutions, such as smartphone apps and wearables, have the potential to be effective in supporting weight loss maintenance if they are evidence-based and offer a choice of behavior change techniques to encourage the use of self-regulatory techniques (eg, self-monitoring). Interventions with human contact are more effective than those that are fully automated [43]. Digital solutions are potentially cost-effective and scalable to large populations, which could engage citizens in health care innovations that are convenient and effective for weight management in the face of limited public budgets [44]. Recent systematic reviews indicate that weight management apps may have positive effects on weight-related outcomes, although the methodological quality of many studies is low and effect sizes are modest [45-47]. It is important to conduct randomized trials of digital technologies for weight loss maintenance and to try to understand the mechanisms by which they may influence weight and health outcomes.

Therefore, effective interventions and commercial programs for weight loss are widely available, but most people regain their lost weight. Currently, few comprehensive solutions exist to help European people manage weight loss maintenance. Current research suggests that the most promising evidence-based behavior change techniques for weight loss maintenance are self-monitoring, goal-setting, action control, building self-efficacy, and intrinsic motivation. Recent research also suggests that stress management and emotion regulation skills are key enablers of relapse prevention and weight regain, but these components have not been formally evaluated through a prospective controlled trial. The Navigating to a Healthier Weight (NoHoW) project tested whether digital delivery of the most promising evidence-based behavior change techniques is effective for weight loss maintenance in a large-scale international 3-center trial of digital tools that implement the most up-to-date behavioral science research. The trial aimed to establish the effectiveness of these tools in supporting weight loss maintenance.

### Study Objectives and Hypotheses

The study’s primary objectives were to evaluate whether using a new digital toolkit was effective for weight loss maintenance by improving (1) self-regulation and motivation, (2) contextual behavioral aspects of emotion regulation, or (3) these factors in combination (motivation and emotion), compared to an active control (generic toolkit content, regular self-weighing, and wearable tracking device [Fitbit] use) in 1627 participants total, at 3 European research centers. The 4-month digital toolkit targeted weight, physical activity, and dietary behaviors through 34 sessions delivered across 15 modules. We hypothesized that the combined intervention would be more effective for weight loss maintenance compared to the self-regulatory or emotion regulatory interventions alone, which individually would also be more effective than the active control group.

Secondary objectives were to determine how the intervention affected health markers (eg, levels of hemoglobin A_1c_ [HbA_1c_]), cholesterol and cortisol levels, and body composition. The study also examined (1) the intervention impact on physical activity, sleep, self-reported dietary intake, depression, anxiety, stress, quality of life, and well-being; (2) potential mediators of weight loss maintenance, such as self-regulation (eg, planning capacity), motivation (eg, autonomous motivation), and emotion regulation processes (eg, self-compassion); (3) quantitative and qualitative assessment of user experience, acceptability, engagement, and dropout; and (4) intervention cost-effectiveness. Secondary outcome analyses (2-4) are being reported in separate publications [48,49].

## Methods

### Study Design

The NoHoW trial was a 3-center (University of Leeds [the United Kingdom], Frederiksberg and Bispebjerg University Hospital [Denmark], and University of Lisbon [Portugal]) 2×2 factorial, randomized, single-blind, controlled trial testing the proof-of-concept of a digital toolkit for weight loss maintenance. A complete description of the trial design can be found elsewhere [50]. In brief, a total of 1627 adults who had achieved ≥5% weight loss in the previous 12 months (initial BMI ≥25 kg/m^2^) were randomized using a 2×2 factorial, single-blind design into four arms: (1) active control (self-monitoring only, including Fitbit activity tracker and smart scale with generic content), (2) self-regulation and motivation (modules targeting goal-setting, action planning, and autonomous motivation), (3) emotion regulation (contextual behavioral strategies such as mindfulness, acceptance, and self-compassion), and (4) combined self-regulation or motivation and emotion regulation. All participants received access to a mobile-enabled digital toolkit integrated with Fitbit Charge 2 and Aria scales. The active intervention lasted 18 weeks during the first 6 months, followed by discretionary use for 12 months. Participants attended clinical investigation days (CIDs) at baseline, 6, 12, and 18 months. The primary outcome was change in weight (kilograms) at 12 months from baseline, with secondary outcomes including body composition, biomarkers (HbA_1c_, lipids, blood pressure, and hair cortisol), physical activity, sleep, dietary intake, psychological mediators, engagement, and cost-effectiveness. Analyses adhered to the prespecified protocol, using intention-to-treat (ITT) principles with multiple imputation for missing data and linear regression models for continuous outcomes.

The toolkit included a set of theoretically informed, evidence-based web-located tools and inputs from digital tracking devices (smart scales and activity trackers) and modules that targeted weight, physical activity, and dietary behaviors. The final toolkit comprised 34 sessions that were distributed through 15 modules and provided active content over a 4-month (18-week) period. The motivation and self-regulation arm consisted of 8 theory-driven modules (17 sessions) targeting self-regulatory behavior change techniques, the emotion regulation arm was presented with 7 theory-driven modules (17 sessions) targeting techniques related to contextual emotion regulation, and the combined arm received the full toolkit (15 modules; 34 sessions). The sessions included a range of implementations, such as videos, testimonies, and questionnaires. Furthermore, the toolkit contained 5 specific data tiles for monitoring weight, steps, healthy eating, mood, and sleep. Full details of the toolkit, its design, and content are described by Marques et al [51]. The toolkit included a mobile-enabled website and tracking technologies: an activity and sleep tracker (Fitbit Charge 2) and a smart wireless body weight scale (Fitbit Aria). The toolkit development followed the Medical Research Council guidance for complex interventions [52], with specific intervention logic models and theory-driven behavior change techniques [51]. The study was conducted between March 2017 and September 2019 in the 3 academic research institutions. The study duration was 18 months with a follow-up at 6, 12 (primary end point, reported in this study), and 18 months after the baseline [50]. Changes in the primary outcome (weight in kilograms) and secondary outcomes (health biomarkers, physical activity, sleep and dietary intake, and self-reported psychological processes) are reported at 12 months from baseline.

**Figure 1.**
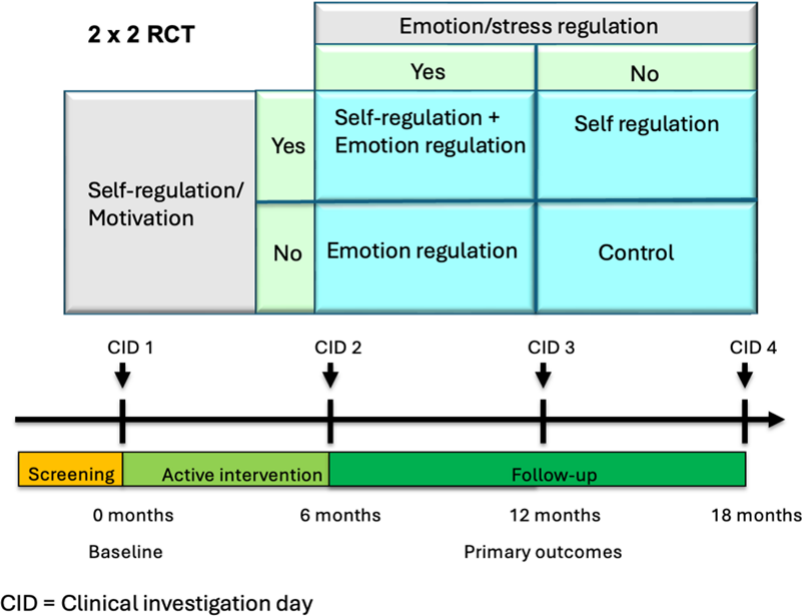
The 2×2 factorial design of the NoHoW trial. CID: clinical investigation day; NoHoW: Navigating to a Healthier Weight; RCT: randomized controlled trial.

In total, 1627 participants (536‐555 per center) were enrolled and randomized into 1 of the 4 intervention arms in a factorial structure. All arms included self-weighing and activity trackers: (1) active control arm (consisting of generic toolkit content), (2) self-regulation and motivation arm, (3) emotion regulation arm, and (4) combined self-regulation or motivation and emotion regulation (motivation and emotion) arm (Figure 1). Participants were allocated to 1 of the 4 treatment arms by adaptive stratified sampling using minimization, where differences in age, weight lost, and current BMI were minimized between treatment arms, which were also balanced for sex. An algorithm was composed and implemented on the trial management website. Staff recruiting participants accessed it there after each individual had been recruited. They were unable to know or predict what allocation the algorithm would select. The nature of the intervention meant that they could not be blinded. The protocol was harmonized across trial centers using Good Clinical Practice guidance, research-grade translation or backtranslation of trial materials, 2 training workshops, and weekly trial management meetings. The final protocol (V2.1 20/09/2017) was approved by the Trial Steering Committee and adhered to the SPIRIT (Standard Protocol Items: Recommendations for Interventional Trials) guidelines [53]. This study was reported using the CONSORT (Consolidated Standards of Reporting Trials) 2025 statement (Checklist 1) [54].

### Ethical Considerations

Ethics approval (ISRCTN88405328) was granted by institutional ethics committees at the Universities of Leeds (17‐0082; February 27, 2017), Lisbon (17/2016; February 20, 2017), and Capital Region of Denmark (H-16030495, March 8, 2017). The protocol was prospectively registered and complied with relevant European Union legislation, international conventions, and declarations relating to ethical research practices 94. All participants were required to sign informed consent. Identifiable information, including name, contact details, and consent form, was obtained as part of the screening process. The identifiable information was pseudonymized via the use of a unique participant study ID. The code link for pseudonymized data is stored for 20 years as a password-protected document accessible only to the research staff. The anonymized data will be kept indefinitely. Participants were compensated for their participation with vouchers (Love to Shop) in the form of a US $20.30 monetary voucher at months 12 and 18. Participants were also allowed to keep their study devices (Fitbit Charge 2 activity tracker and Fitbit Aria scales).

### Study Population

The trial examined weight loss maintenance in those who had lost ≥5% of their weight during the previous 12 months. Participants were adults (≥18 years) with a pre–weight-loss BMI ≥25 kg/m^2^, who had achieved and maintained ≥5% weight loss within the past 12 months. Eligibility required access to a smartphone or computer with internet, ability to use standing scales (≤150 kg), and provision of informed consent. Exclusion criteria included weight loss due to illness or surgery, pregnancy or breastfeeding, eating disorders, certain unstable medical conditions, recent type 1 diabetes diagnosis, inability to communicate in English, Danish, or Portuguese (at respective centers), participation in conflicting studies, extensive planned travel, or cohabitation with another trial participant. Prior to randomization, potential participants were asked to provide documented verification (by a health professional, weight loss counselor or friend, weight loss program record booklet, diary or smartphone app, or before or after photographs) that they had achieved a clinically significant weight loss of >5% during the previous 12 months. The complete inclusion or exclusion criteria for the trial, recruitment, interventions, assessment, and study procedures are described in a previously published protocol paper [50]. There were no deviations from the main details stated in the protocol. There were minor deviations for a few participants (eg, hair not being cut correctly), which were all recorded and not considered to have any significant impact on the results.

Center-specific recruitment strategies were adopted for 12 months (March 2017-March 2018) and included commercial weight loss programs (the United Kingdom, Slimming World), the Copenhagen Municipality weight management services, dieticians from the Danish Association for Dieticians and commercial slimming companies, registered clinical dieticians or nutritionists who provide weight management services in Lisbon, leisure centers, and local or national media coverage and advertisements. All participants were directed to country-specific recruitment websites and completed an online eligibility screener using Qualtrics. The eligibility screener categorized respondents as eligible, potentially eligible (waitlist), and ineligible. Eligible individuals were contacted for a telephone screening interview, provided with study information, medical history questions, and the Physical Activity Readiness Questionnaire [55]. Eligible participants were invited to a CID, where informed consent was obtained by research staff before randomization (Multimedia Appendix 1).

Each participant attended 4 CIDs at baseline, 6, 12, and 18 months. The purpose of these visits was to conduct standardized assessments and ensure protocol adherence. At the baseline CID, informed consent was obtained, eligibility confirmed, and participants were randomized and trained on the use of the digital toolkit and Fitbit devices. Subsequent CIDs included reconsent, collection of primary and secondary outcome measures (eg, weight, body composition, blood pressure, biomarkers, and psychometric questionnaires), and troubleshooting any technical issues. Participants were reconsented before each subsequent visit and excluded if they became ineligible.

### Measurements

A full description of trial measures and associated references (including mediators and moderators of any trial effects) is described in a previous publication [50]. Prespecified cardiometabolic secondary outcomes (waist circumference, fat mass, fat-free mass, HbA_1c_, blood lipids, blood pressure, resting heart rate, and hair cortisol) are analyzed and reported in this paper. All other secondary outcomes (physical activity, sleep, dietary intake, eating behavior, well-being, and psychosocial variables) results are available in Tables S3 and S4 in Multimedia Appendix 1. All measures were taken at the intervention centers by the trial team. The research team was not blinded to the assignment.

Data were collected in a variety of ways and stored centrally in a data hub. Measurements on each CID were entered in commercial trial management software (Easytrial; EasyTrial ApS) and subsequently uploaded to the data hub. User engagement, activity, and comments were recorded by the toolkit and stored on the data hub. Dietary intakes were recorded using Intake24 [56] and periodically downloaded from their servers and uploaded to the data hub. A similar process was followed with questionnaire data, which were recorded online by Qualtrics. Wearable devices and weighing scales streamed data to Fitbit servers, and an application programming interface running on the data hub pulled data once every day from the Fitbit data storage server and added it to tables in the database. These tables were accessible to trial staff who could thereby monitor compliance.

### Primary Outcome

Body weight (±0.1 kg) was measured using a Seca 704s instrument (SECA) in participants wearing light clothing. Participants were asked to self-weigh themselves at least twice weekly, in the morning after voiding and before eating using the provided Fitbit Aria scales for 18 months.

### Secondary Outcomes

#### Body Composition and Height

Multifrequency whole bioimpedance spectroscopy was measured by ImpediMed SFB7, which measures impedance over a spectrum of frequencies for the estimation of body composition. Hanai mixture theory equations and standard resistivity constants [57] were used to calculate fluid volumes to estimate total body water and hence fat and fat-free mass. Height (±0.1 cm) was measured with participants barefoot, using a Seca 704s instrument (SECA).

#### Waist and Hip Measurements

Waist and hip measurements were taken according to the World Health Organization’s guidance. A tape measure was used to record the hip and waist circumference to the nearest 0.1 cm [58].

#### Biomarkers

##### HbA_1c_ and Cholesterol

Fasted capillary blood samples were collected to determine HbA_1c_ (mmol/mol, %), estimate average glucose (mol/L), and calculate full lipid profiles, including total cholesterol, low-density lipoprotein, high-density lipoprotein (HDL), triglycerides, non-HDL, and cholesterol or HDL (mmol/L) assayed using a bench-top analyzer (Alere Afinion AS100 Analyzer) [59].

##### Systolic and Diastolic Blood Pressure and Resting Heart Rate

Systolic and diastolic blood pressure and resting heart rate were measured with the participant at rest in the sitting position (Microlife BP A2 Basic, Gentle Technology). Values were taken as the average of 3 measurements.

##### Cortisol

Hair cortisol was included as a measure of chronic stress using hair samples following a previously described protocol [60]. Hair samples were cut from the posterior vertex as close to the scalp as possible. Between 10 and 30 mg of the hair from 2 cm closest to the scalp was cut into small pieces and dissolved in 1 mL methanol and incubated at ultrasound sonication for 30 minutes, followed by 18 hours at 52 °C in a shaking incubator (300 rpm). Hair samples were not collected among participants with less than 2 cm of hair. The methanol was transferred to a new tube and evaporated to dryness under a stream of nitrogen at 45 °C. Dried samples were stored at −20 °C until analysis. Before analysis, samples were redissolved in 500 µL phosphate-buffered saline at pH 8 and centrifuged at 2000 rpm for 2 minutes. Reconstituted samples were analyzed with a cortisol enzyme-linked immunosorbent assay (Alpco).

### Physical Activity

Minute-by-minute physical activity data and heart rate were measured by the Fitbit Charge 2 for the study duration. Data were used from 2 weeks after randomization for baseline and for the 8 weeks prior to the 12-month point for the trial outcome. Physical activity was also self-reported at 0, 6, 12, and 18 months (8-week intervals) using the International Physical Activity Questionnaire [61] and the Activity Choice Index [62].

### Sleep Quality and Quantity

The Fitbit Charge 2 estimates sleep quantity (hours/minutes) and quality (stages of sleep) using proprietary algorithms. Data were collected daily throughout the trial.

### Dietary Intake and Eating Behavior

Four consecutive 24-hour web-based dietary recalls, including at least 1 weekend day, were collected within 7 days of each CID visit using Intake24 [56], and the average daily intake of energy and macronutrients was calculated. Intake24 is an open-source self-completed computerized dietary recall system based on a multiple-pass 24-hour recall. The online system offers similar data quality to interviewer-led recalls at a significantly lower cost. Dietary information was collected at baseline and the 12-month follow-up using a 4-day, 24-hour dietary recall via the online platform Intake24, which included at least 1 weekend day [50,56]. Not all participants completed all four 24-hour recalls [63]. The recorded weights of foods and beverages were converted into energy and nutrient intakes for each item and then summed to yield total 24-hour energy and nutrient intakes for each participant on each recall day. Daily macronutrient intakes were estimated using a country-specific food composition database [50]. The primary exposures were the proportions of total energy intake derived from protein, fat, and carbohydrate at baseline, analyzed as continuous variables (percentage of total energy). Questionnaires were completed by the participants at home using Qualtrics. The following measures were collected at the time of each CID at 0, 6, 12, and 18 months:

- Three-Factor Eating Questionnaire-51: The Three-Factor Eating Questionnaire includes 51 items measuring eating disinhibition, susceptibility to hunger, and dietary restraint [64].
- Binge Eating Scale [65]: This includes 16 items and assesses the behavioral, cognitive, and emotional dimensions of binge eating symptomatology.
- Intuitive Eating Scale-21: This is a 21-item scale that assesses 3 subscales of intuitive eating: eating for physical rather than emotional reasons, unconditional permission to eat, and reliance on hunger and satiety cues [66].
- Well-Being and Quality of Life (EQ-5D-5L): EQ-5D-5L is a measure of generic health-related quality of life including mobility, self-care, usual activities, pain or discomfort, and anxiety or depression [67].
- Warwick-Edinburgh Well-Being Scale: This is a 14-item single-factor scale, which assesses mental well-being [68].

### Sample Size Calculation

Power calculations were based on the primary outcome (weight change) in previous trials [69] at 12 months and also conducted for the secondary outcome change in HbA_1c_. To detect a difference between treatment arms of >1.5 kg body weight with a Cohen *d* value of 0.25 for 80% power, comparing more than 2 groups required a sample size of 250 per trial arm. To detect an effect size of 0.25 SD units for HbA_1c_, 245 participants in each trial arm gave 80% power at 5% significance. Assuming a 38% dropout, a sample size of 1600 (533 per center) was needed to achieve a sample of 1002 (334 per center, ~250 per trial arm) participants at 12 months. These choices were informed by previous trials in which the investigators had been involved [69], and we estimated dropout rates from these, though we note that dropout in NoHoW was less than we predicted. A difference of 1.5 kg was our experienced judgment on the magnitude of difference in effect that would be relevant to health.

### Statistical Analysis

#### Primary Analysis (ITT)

The prespecified primary analysis evaluated intervention effects on 12-month weight change (kilograms) using an ITT framework. For the primary outcome, we also reported the results on 2 other study subsets, the completers (those who had measurements at 12 months) and the per-protocol sample (those who had measurements at 12 months and who also completed at least 80% of the toolkit modules for their assigned trial arm).

Baseline data were summarized by group and sex, and the significance of these factors was assessed by a linear model, which also included terms for center, age group, and pretrial weight loss group. Statistical analyses were carried out using linear models, where the response was the change in outcome value from baseline to 12 months. Explanatory terms were center, sex, motivation intervention, emotion intervention, and 2-way interactions between the terms motivation and emotion. Age group, BMI group, and pretrial weight loss group were included as additional covariates. Significance was assessed using type II sums of squares to maximize power to detect main effects in the absence of significant interactions in an ANOVA of the linear model. Means presented are marginal means estimating the effects in a population, in which other factors are equally balanced between trial arms.

ITT requires imputation of outcome data for those who had dropped out. An assumption of missingness at random did not appear valid (Little [70] test of whether data are missing completely at random gave *P*<.001), and so standard multiple imputation was unsuitable. Carrying forward, the last observation would assume no weight regain in those who drop out, which appeared likely to treat dropouts as having an unreasonably optimistic outcome. We therefore used the most conservative assumption that dropouts had regained all weight lost before the trial, that is, we carried forward the highest weight in the 12 months before CID 1 (baseline observation carried forward). Weight lost before the trial was available, but other outcomes were not. As using CID 1 measures for baseline carried forward would appear optimistic for these outcomes in dropouts, and they were not available before the pretrial weight loss, we imputed values by predicting them from the participant characteristics and their imputed weight regain. Multiple imputation, using the R library “mice,” was used to account for the uncertainty in this prediction. Data analysis was carried out using R (version 3.5; R Foundation for Statistical Computing).

#### Subgroup Analyses (Prespecified: Men and Women)

Prespecified sex subgroup analyses were conducted by fitting the same models separately in men and women to assess whether intervention effects differed by sex. These analyses are reported after the primary ITT results, and tables and figures are placed in Multimedia Appendix 1.

#### Sensitivity Analyses (Completers and Per-Protocol)

Two sensitivity analyses were conducted to examine the robustness of the primary ITT findings. This includes a completer (participants with observed 12-month weight) and per-protocol (participants with observed 12-month weight and ≥80% completion of assigned toolkit modules in their randomized arm) analysis. These analyses were prespecified in the published protocol [50].

#### Exploratory Analyses (Post Hoc: Engagement or Compliance)

Exploratory post hoc regressions assessed associations between engagement metrics and weight change. Predictors included toolkit compliance (percentage of assigned modules completed within the intervention arm), Fitbit compliance (number of days with >20 hours of wear recorded), and self-weighing compliance (number of weeks with ≥2 weighings on the provided smart scale). These exploratory analyses were not part of the prespecified primary analysis.

## Results

### Participants

**Figure 2.**
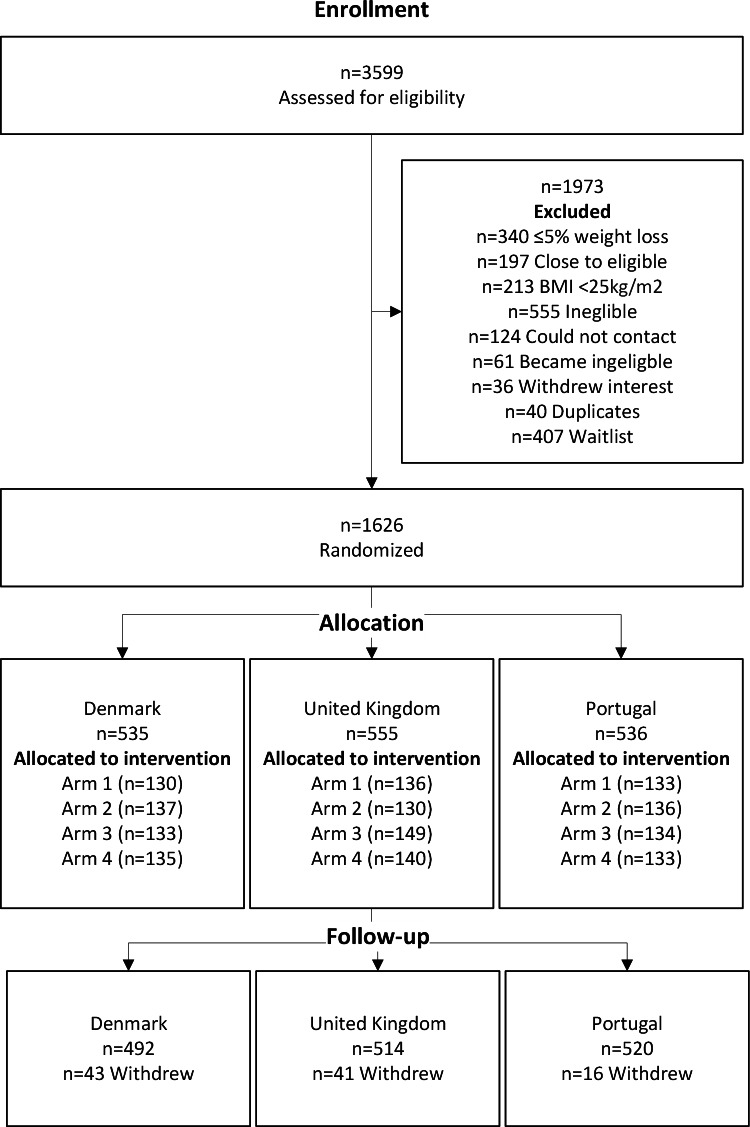
CONSORT (Consolidated Standards of Reporting Trials) diagram showing participant flow through the NoHoW 2×2 factorial randomized controlled trial of a digital toolkit for weight loss maintenance. NoHoW: Navigating to a Healthier Weight.

The sample consisted of 1117 (68.7%) women and 510 (31.3%) men (Figure 2). In the 12 months prior to randomization, the women lost ~11% (10.8 kg), and the men lost ~12% (10.7 kg) of their weight (*P*<.001). The mean age was 44.5 (SD 11.9) years, weight was 84.7 (SD 17.2), and BMI was 29.7 (SD 5.5) kg/m^2^. At the 12-month follow-up (CID3), 364 of 1627 (22.4%) of participants had missing data.

At baseline, the majority of participants had a BMI of either 25‐29.9 kg/m^2^ (689/1627, 42.3%) or 30‐34.9 kg/m^2^ (398/1627, 24.5%). In total, 1594 participants provided data on employment. Most (n=1089, 68.3%) were employed full-time, 8.3% (n=132) were employed part-time, 8.7% (n=139) were retired, 6.3% (n=100) were students, 2.4% (n=38) were unemployed, 0.9% (n=14) were self-employed, and 0.5% (n=8) were caregivers. Most (496/555, 89.4%) UK participants described themselves as White British; 97.2% (521/536) participants from Denmark described themselves as Danish; and 69.78% (374/536) participants from Portugal described themselves as Portuguese. The majority (n=1384, 85.1%) of the sample were educated beyond secondary-level education (eg, degree level). In total, 43% (n=700) of participants were married. The median household income for the Danish and UK samples fell into the same category as their national average (range of €22,400-€53,713 [US $25,847.99-$61,980.95]). For the Portuguese sample, median household income fell below their national average (€12,000‐€18,000 [US $13,847.14-$20,770.71]). Mean baseline values of the study variables for all participants and per center are reported in Table 1.

**Table 1.** Baseline characteristics of participants in the NoHoW^a^ trial, a 2×2 factorial randomized controlled trial of a digital weight loss maintenance intervention in European adults^b^.

		All participants				All	*P* value
		Control	Motivation	Emotion	Motivation + emotion		
Center, n (%)							.98
	Copenhagen	131 (32.8)	137 (34)	133 (32)	135 (33.1)	536 (32.9)	
	Lisbon	133 (33.2)	136 (33.7)	134 (32.2)	133 (32.6)	536 (32.9)	
	Leeds	136 (34)	130 (32.3)	149 (35.8)	140 (34.3)	555 (34.1)	
Sex, n (%)							>.99
	Female	276 (69)	276 (68.5)	285 (68.5)	280 (68.6)	1117 (68.7)	
	Male	124 (31)	127 (31.5)	131 (31.5)	128 (31.4)	510 (31.3)	
Age group (years), mean (SD)							.47
	18‐29	54 (13.5)	46 (11.4)	58 (13.9)	41 (10)	199 (12.2)	
	30‐49	219 (54.8)	240 (59.6)	226 (54.3)	233 (57.1)	918 (56.4)	
	50‐85	127 (31.8)	117 (29)	132 (31.7)	134 (32.8)	510 (31.3)	
Pretrial weight loss (kg), mean (SD)		9.6 (7.95)	10.68 (9.92)	10.07 (8.9)	9.84 (8.12)	10.05 (8.75)	.44
Body composition (kg), mean (SD)							
	Weight (kg)	84.96 (17.46)	84.62 (16.89)	84.18 (17.06)	85.45 (17.66)	84.8 (17.26)	.75
	Fat-free mass (kg)	57.44 (11.51)	57.19 (11.61)	56.85 (11.4)	57.49 (11.31)	57.24 (11.45)	.85
	Fat mass (kg)	27.3 (10.7)	27.27 (10.07)	27.07 (10.15)	27.86 (10.93)	27.37 (10.46)	.74
Waist circumference (cm), mean (SD)		93.97 (14.89)	93.6 (13.67)	93.94 (13.88)	95.38 (14.69)	94.22 (14.29)	.30
Biomarkers, mean (SD)							
	HbA_1c_^c^ (mmol mol)	33.04 (5.71)	33.09 (6.47)	32.87 (5.84)	33.04 (4)	33.01 (5.58)	.95
	HbA_1c_ (%)	5.19 (0.52)	5.2 (0.59)	5.18 (0.53)	5.19 (0.37)	5.19 (0.51)	.96
	Total cholesterol (mmol/L)	4.78 (0.99)	4.9 (1.05)	4.9 (1)	4.99 (1.09)	4.89 (1.04)	.06
	LDL^d^ cholesterol (mmol/L)	2.65 (0.84)	2.74 (0.87)	2.76 (0.85)	2.84 (0.92)	2.75 (0.87)	.03
	HDL^e^ cholesterol (mmol/L)	1.58 (0.41)	1.58 (0.42)	1.57 (0.41)	1.57 (0.4)	1.57 (0.41)	>.99
	Triglycerides (mmol/L)	1.28 (0.87)	1.35 (0.93)	1.27 (0.81)	1.34 (1.01)	1.31 (0.91)	.54
	Systolic blood pressure	120.87 (14.34)	120.68 (14.74)	122.49 (15.75)	122.69 (14.04)	121.69 (14.75)	.10
	Diastolic blood pressure	75.95 (8.93)	75.81 (8.96)	76.62 (9.34)	77.51 (9.31)	76.48 (9.16)	.03
	Heart rate (bpm)	65.81 (10.08)	65.91 (11.01)	65.58 (10.54)	66.76 (11.06)	66.01 (10.68)	.41
	Hair cortisol (log)	3.98 (1.14)	3.92 (1.2)	3.99 (1.2)	3.99 (1.25)	3.97 (1.2)	.90

a NoHoW: Navigating to a Healthier Weight.

b Characteristics include demographics, anthropometry, and clinical markers at baseline across the 4 intervention groups (control, motivation, emotion, and motivation+emotion). Study population: adults with ≥5% weight loss in the previous 12 months, recruited in the United Kingdom, Denmark, and Portugal.

c HbA_1c_: hemoglobin A_1c_.

d LDL: low-density lipoprotein.

e HDL: high-density lipoprotein.

### Primary Analysis (ITT)

There was an overall tendency for pretrial weight loss to be regained on average (Table 2). Participants were assigned to the following weight change categories: >−3% weight loss (weight loser), between −2.99% and +2.99% (weight maintainer), and >+3% weight gain (weight gainer) [71]. The results showed that 19% (302/1627) of participants continued to lose weight, 32% (302/1627) maintained weight, and 49% (811/1627) regained some weight (at ±3% weight change). When we investigated weaker assumptions, such as regain of a proportion of this weight loss, the main assessment of the intervention effects was similar, although absolute estimates of weight regain were lower in all arms (Table 2). Table 2 shows body composition and cardiometabolic biomarkers at 12 months by intervention arm for the whole sample. The motivation arm, the emotion arm, and the combined emotion and motivation arms did not significantly affect weight outcomes compared to the active control.

**Table 2.** Primary and prespecified secondary cardiometabolic outcomes at 12 months in the NoHoW^a^ 2×2 factorial randomized controlled trial^b^.

		Control, mean (95% CI)	Motivation, mean (95% CI)	Emotion, mean (95% CI)	Motivation + emotion	Motivation, *P* value	Emotion, *P* value	Motivation × emotion, *P* value
Body composition								
	Weight change (kg)	4.58 (3.41 to 5.75)	4.01 (2.82 to 5.2)	5.09 (3.92 to 6.26)	4.57 (3.38 to 5.76)	.49	.38	.95
	Fat-free mass (kg)	4.58 (3.42 to 5.75)	4.47 (3.43 to 5.51)	4.62 (3.61 to 5.63)	4.69 (3.53 to 5.84)	.86	.81	.73
	Fat mass (kg)	2.88 (1.7 to 4.07)	2.58 (1.57 to 3.58)	3.32 (2.31 to 4.33)	2.88 (1.76 to 4)	.29	.79	.78
	Waist circumference (cm)	4.03 (2.97 to 5.09)	3.41 (2.33 to 4.49)	4.24 (3.04 to 5.45)	3.89 (2.73 to 5.04)	.39	.62	.80
Biomarkers								
	HbA_1c_^c^ (mmol mol)	1.46 (0.96 to 1.96)	1.3 (0.84 to 1.77)	1.54 (1.07 to 2.01)	1.4 (0.91 to 1.88)	.43	.28	.83
	HbA_1c_ (%)	0.15 (0.12 to 0.18)	0.13 (0.1 to 0.17)	0.15 (0.11 to 0.18)	0.13 (0.1 to 0.17)	.31	.69	.91
	Total cholesterol (mmol/L)	0.19 (0.08 to 0.29)	0.23 (0.13 to 0.34)	0.26 (0.14 to 0.38)	0.29 (0.17 to 0.42)	.37	.12	.96
	LDL^d^ cholesterol (mmol/L)	−0.05 (−0.2 to 0.11)	−0.01 (−0.12 to 0.1)	0.03 (−0.09 to 0.14)	0.05 (−0.06 to 0.16)	.67	.06	.72
	HDL^e^ cholesterol (mmol/L)	0.1 (0.04 to 0.16)	0.11 (0.06 to 0.15)	0.08 (0.04 to 0.13)	0.11 (0.06 to 0.16)	.66	.92	.55
	Triglycerides (mmol/L)	0.3 (0.14 to 0.47)	0.25 (0.11 to 0.4)	0.29 (0.14 to 0.45)	0.23 (0.08 to 0.38)	.47	.66	>.99
	Systolic blood pressure (mm Hg)	1.97 (0.4 to 3.55)	1.44 (−0.3 to 3.19)	1.58 (−0.11 to 3.27)	2.55 (0.95 to 4.14)	.58	.27	.15
	Diastolic blood pressure (mm Hg)	1.48 (0.17 to 2.79)	0.54 (−0.75 to 1.83)	1.11 (−0.11 to 2.33)	1.06 (−0.07 to 2.2)	.55	>.99	.26
	Heart rate (bpm)	2.05 (0.62 to 3.48)	2.13 (0.75 to 3.5)	3.06 (1.62 to 4.51)	2.15 (0.52 to 3.79)	.46	.31	.39
	Hair cortisol (log)	0.22 (0.01 to 0.43)	0.24 (0.02 to 0.46)	0.26 (0.04 to 0.48)	0.22 (0.02 to 0.42)	.70	.59	.77

a NoHoW: Navigating to a Healthier Weight.

b Values are mean (95% CI) change from baseline to 12 months for waist circumference, fat mass, fat-free mass, HbA_1c_, blood lipids, blood pressure, resting heart rate, and hair cortisol across intervention arms. *P* values reflect factorial main effects for the motivation component, emotion regulation component, and their interaction (motivation×emotion). Population: adults enrolled in the NoHoW digital weight-loss-maintenance randomized controlled trial across 3 European centers.

c HbA_1c_: hemoglobin A_1c_.

d LDL: low-density lipoprotein.

e HDL: high-density lipoprotein.

### Subgroup Analyses (Prespecified: Men and Women)

Table S1 in Multimedia Appendix 1 shows sex-stratified body composition and cardiometabolic biomarkers at 12 months by intervention arm. Sex differences showed that a small effect of the motivation arm was significant in men only for the ITT, per-protocol, and completer populations. The analysis was factorial, looking at the effect of motivation and the effect of emotion and interactions. The effect of motivation was slightly significant, but we could not conclude that the interaction was significant; that is, we could not conclude that we had enough evidence to say motivation and emotion were more effective than motivation.

Figure S1 in Multimedia Appendix 1 shows longitudinal changes in body weight by trial arm (control, self-regulation and motivation, emotion regulation, and self-regulation and motivation plus emotion regulation combined) for men and women. Figure S2 in Multimedia Appendix 1 shows the percentage of the ITT population gaining, losing, or maintaining pretrial weight loss by trial arm (control, motivation, motivation and emotion, and emotion). Percentage weight change was calculated as (CID3 kg–CID1 kg)/CID1 kg×100. The weight category was then calculated from the percentage weight change.

### Sensitivity Analyses (Completers and Per-Protocol)

There was an overall tendency for pretrial weight loss to be regained on average (Table 2). For those who completed measures (ie, the per-protocol sample) at 12 months, men regained 0.14 kg (0.27%) of the weight they lost, and women regained 0.54 kg (0.85%) of the weight they lost. However, there was substantial variation between participants. For the per-protocol population, 24% (302/1263) continued to lose weight, 41% (514/1263) maintained weight, and 35% (447/1263) regained weight (Table 3). See Table S2 in Multimedia Appendix 1 for the sex-stratified sensitivity analyses of 12-month weight change (kilograms) for the ITT, per-protocol, and completer populations by intervention arm.

**Table 3.** Sensitivity analyses of 12-month weight change (kilograms) in the NoHoW^a^ trial, including intention-to-treat (ITT), completer, and per-protocol (PP) populations^b^.

	Control, mean (95% CI)	Motivation, mean (95% CI)	Emotion, mean (95% CI)	Motivation+emotion, mean (95% CI)	Motivation, *P* value	Emotion, *P* value	Motivation×emotion, *P* value
ITT	4.58 (3.41 to 5.75)	4.01 (2.82 to 5.2)	5.09 (3.92 to 6.26)	4.57 (3.38 to 5.76)	.49	.38	.95
PP	0.46 (−0.85 to 1.78)	−0.74 (−2.24 to 0.76)	−0.56 (−2.12 to 1)	−1.63 (−3.37 to 0.12)	.05	.39	.89
Completer	0.85 (−0.25 to 1.95)	0.39 (−0.72 to 1.51)	0.94 (−0.17 to 2.04)	0.46 (−0.67 to 1.58)	.44	.94	.97

a NoHoW: Navigating to a Healthier Weight.

b Values are mean (95% CI) change in body weight from baseline to 12 months for each intervention arm. *P* values reflect factorial main effects for motivation, emotion regulation, and their interaction (motivation×emotion). The PP population includes participants completing ≥80% of their assigned intervention modules.

**Figure 3.**
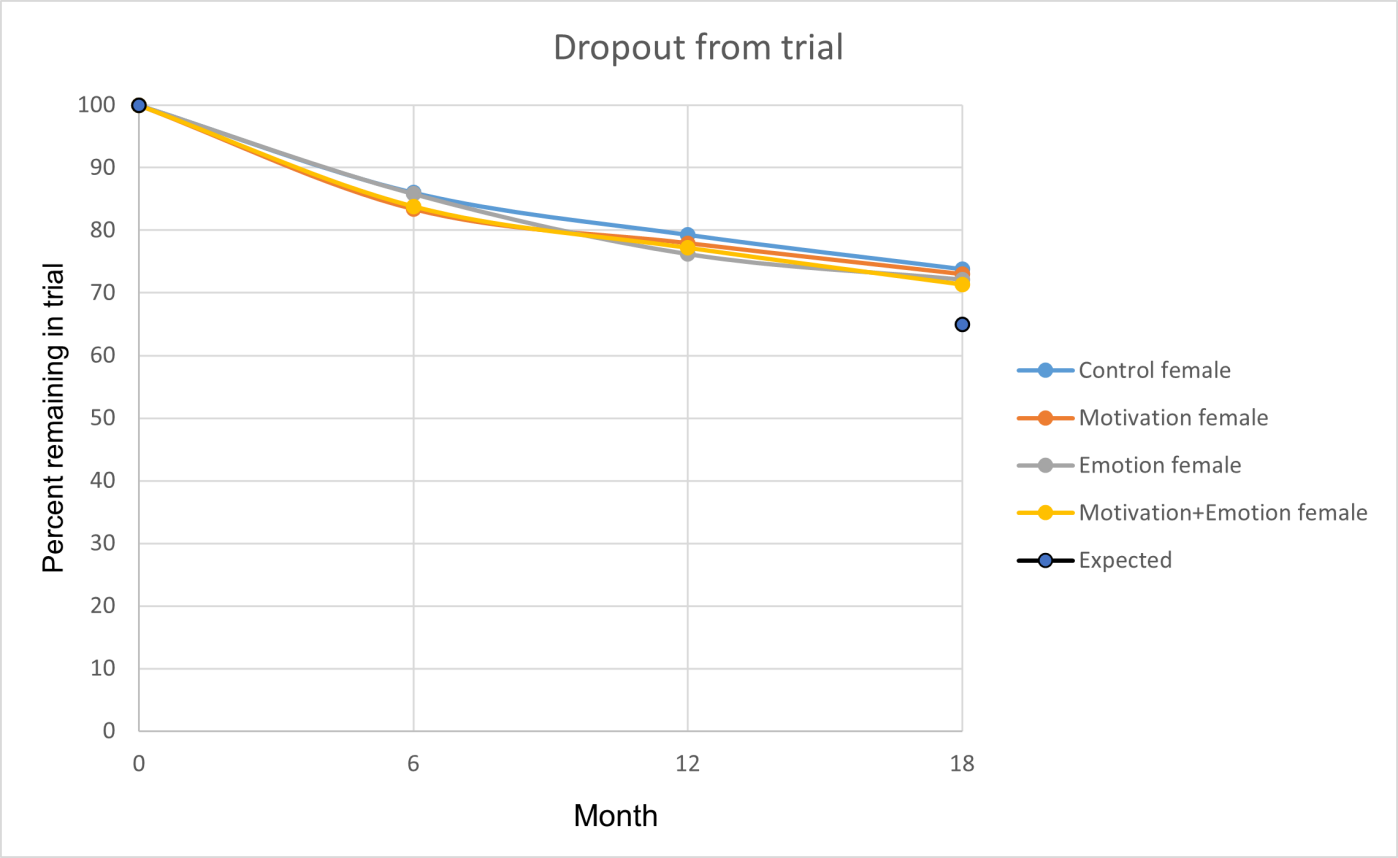
Retention of participants over time across intervention arms in the NoHoW 2×2 factorial randomized controlled trial. The figure displays cumulative dropout from baseline to 12 months and to 18 months for each intervention group (control, motivation, emotion, and motivation+emotion). Values represent the proportion retained at each follow-up time point. The purple dot at month 18 indicates an assumed dropout of 38% estimated from previous randomized controlled trials. NoHoW: Navigating to a Healthier Weight.

Figure 3 shows that retention of participants in the trial was high and greater than assumed for the power calculations. By the 12-month primary outcome, over 77% (1263/1627) of the ITT sample was still engaged in the intervention, and by 18 months, over 71% of the ITT sample was still engaged in the intervention for all trial arms.

### Exploratory Analyses (Post Hoc: Engagement or Compliance)

Table 4 shows univariate and multivariate regression analysis using toolkit compliance, Fitbit compliance, and self-weighing as predictors of weight outcomes. The univariate models for toolkit use, Fitbit compliance, and self-weighing all showed small predictive effects on weight loss, but in multivariate models, only self-weighing remained as a significant predictor independently of the other measures. However, the proportion of variance explained remains small.

**Table 4.** Exploratory associations between digital engagement metrics and 12-month weight change in the NoHoW^a^ randomized controlled trial^b^.

Predictor	Univariate models			Multivariate models		
	Coefficient	SE	*P* value	Coefficient	SE	*P* value
Toolkit use compliance (percentage)	−0.0259	0.0076	.001	−0.0113	0.0080	.16
Fitbit use compliance (number of days>20 hours use)	−0.0048	0.0014	.001	−0.0004	0.0017	.80
Self-weighing compliance (number of weeks with ≥2 weighings)	−0.0905	0.0109	<.0001	−0.0860	0.0135	<.0001

a NoHoW: Navigating to a Healthier Weight.

b Univariate and multivariable linear regression models examined associations between toolkit compliance, Fitbit wear-time compliance, and self-weighing frequency with weight change from baseline to 12 months. These analyses were post hoc and not prespecified.

## Discussion

### Principal Findings

NoHoW was the first project to develop and formally evaluate (via a randomized controlled trial [RCT]) a digital toolkit combining continuous tracking of energy balance behaviors and body weight with theoretically informed, evidence-based digital interventions targeting self-regulation and motivation, and emotion regulation in a 2×2 factorial design, to target long-term weight management.

This study was designed to test three hypotheses that (1) motivation and self-regulation of energy balance behaviors improve longer-term weight outcomes, (2) emotion regulation strategies help prevent weight relapse, and (3) there is an additive effect between emotion regulation and self-regulation or motivation. In the primary ITT analysis for all participants, none of the intervention arms differed significantly from the active control at 12 months. Exploratory subgroup analyses suggested a small effect of the motivation intervention among men, but this effect was not clinically meaningful and did not alter the overall null findings of the primary analysis. This intervention arm was also the arm in which intervention completion (but not necessarily the number of visits to the toolkit) had the highest correlation with weight loss [48]. The 2×2 factorial design was well-powered to detect intervention effects; yet, the interventions showed no detectable effect on the primary outcome and no meaningful effect on prespecified cardiometabolic secondary outcomes.

Although subgroup analyses indicated a small effect in men, these analyses were exploratory and should be interpreted with caution. These findings were not consistent with engagement data and, importantly, do not outweigh the primary ITT result, showing no intervention effect in the overall sample. Men may have been more engaged with the process-based intervention content, but user metrics for the digital intervention do not support this notion. Indeed, focusing on toolkit engagement (compliance) alone, there was a negative correlation between use metrics and weight change (all *P*<.05). However, multiple comparison adjustment would weaken these statistics. Measured use of the toolkit components suggested that men tended to use the toolkit less than women and that use was lower in the motivation arm (in which weight loss was greatest in men only), which does not support the hypothesis that compliance or engagement with the motivation arm of the toolkit explains the slightly greater weight loss in men.

### Explaining the Negligible Effect of the Digital Intervention for Weight Loss Maintenance

Possible explanations for the lack of intervention effect may relate to the fact that the intervention was digital, that the toolkit content and dose per se (ie, the logic models) were not as effective as hypothesized (ie, those mechanisms are just not very effective and the dose was not sufficient), or that participants did not engage with the intervention (ie, this implies that the toolkit’s design may not have adequately supported the delivery of its proposed mechanisms of action, which, if implemented more effectively, might have yielded the expected effects). Evidence-based behavior change interventions tend to show small effect sizes anyway [18-20,25,72,73]. In fact, the evidence tends to point in the direction that the most salient component of this trial associated with weight change was self-weighing.

The participant user engagement and experience evaluation of the content of the toolkit itself was revealing [48]. In this study, the actual use of the toolkit during 18 weeks of the active intervention was relatively high and comparable to other digital health interventions, and as with similar interventions, tended to decay with time [48]. The use declined rapidly during the period of discretionary use. Weight outcomes were influenced by the degree of engagement with the study, as has been shown previously [74]. Weight outcomes were also weakly correlated with the use of the digital intervention content, but correlations between weight change from baseline to 12 months and use metrics were small and not consistent with the small differences in weight outcomes across trial arms (Table 2). While participants had some reservations about the content and ease of use of the toolkit, it does not appear that the degree of engagement, as measured by actual use of the toolkit, is a viable explanation for the general lack of between-arm effects of the intervention (Table 4).

Usability was rated as satisfactory, whereas acceptability was modest (between 3 and 4 on a 5‑point scale). Both declined across all trial arms over the course of the study. Qualitative data showed that these perceptions of the toolkit were similar across centers and intervention arms [48]. Participants appreciated support for self-monitoring of weight, sleep, and activity, all of which were derived from the Fitbit devices. Participants also appreciated the content of the toolkit based on self-regulation of behavior and emotion regulation. They associated their decreased toolkit use with frustrations in design that hindered its use, for example, the log-in process as well as time commitment and frustrations using the toolkit on mobile phones [48]. Participants also tended to compare the design aspects of the toolkit unfavorably to the Fitbit app and other commercial offerings [48]. Such offerings are also regularly updated, while the NoHoW toolkit was static for 18 months to ensure that the trial was testing the same intervention in all participants. Thus, the user acceptability and experience data offer important insights into the limited impact of the digital intervention on weight outcomes. The intervention appears to have been less preferred than the Fitbit app, and intervention effects were very small. It does not matter how evidence-based or research-informed an intervention is if it is deemed to be difficult or even mediocre to use compared to other offerings available.

Thus, while it is important that digital behavior change interventions need to be developed with an interdisciplinary team and that the content, delivery, and structure are informed by theory and evidence [51], there are other important design aspects that could be addressed to improve the effect sizes of interventions such as the NoHoW toolkit. The NoHoW toolkit was meticulously developed with respect to (1) state-of-the-art theories (self-regulation theory, self-determination theory, and aspects of emotional regulation theory), (2) integration of consumer physical activity and weight tracking devices, (3) web-design expertise, (4) some user testing, and (5) preliminary mixed methods research. However, the delivery of the digital intervention may have benefited from greater involvement from human-computer interaction experts and co-design with prospective users together with commercial partners. There is some evidence that combining digital and human support may improve effective engagement with digital behavior change interventions, although more evidence is needed to configure the most effective combinations [75,76]. There is some evidence for similar effects in relation to mental health [77]. Such partners may have offered resources and infrastructure to produce an offering that motivates engagement and reinforces the practice of behavior change techniques using refreshed content, which may have improved outcomes. Due to resource constraints, these design aspects were limited in this trial. Involvement of health care professionals and engagement with social networks as a part of the intervention may improve some outcomes [78,79]. Using optimization designs for iterative development and testing of smaller components (eg, clusters or individual behavior change techniques) could also be beneficial to identify the combination of components that could work better together [80]. The NoHoW trial deliberately excluded interactions with health care professionals and social networks in order to test the effect of a digital-only intervention. A constraint of an RCT design is the fixed nature of the intervention during the trial. This approach precludes iterative development, testing, and ongoing content and design updates, the lack of which disappointed participants who are used to such features of commercial apps [48]. These factors should be taken into account in developing future digital interventions for weight management.

### Is There Evidence That Fitbit Use Predicted Weight Loss Maintenance?

A possible factor (point 2 in the design specifications listed earlier) that may have limited the effect sizes observed in this study is the use of an active control, in which participants each received and used a Fitbit Charge 2 activity tracker and a set of Fitbit Aria wireless scales. All participants also had access to the Fitbit smartphone app. The use of trackers and scales was high [81,82]; therefore, it is likely that these represented an “intervention within an intervention.” Ideally, it would have been better to conduct a 2×2 plus 1 trial, in which there was a genuine “no intervention” control, but resources did not permit this option. In regression models, weight change was significantly predicted by all of toolkit compliance, self-weighing compliance, and Fitbit use compliance. However, when all 3 were included in a model, only self-weighing compliance was significant; that is, there was little evidence that the toolkit had any effect over and above using the Fitbit devices alone.

### Comparison to Other Trials Using Behavior Change Approaches for Longer-Term Weight Management

At present, systematic reviews and meta-analyses show the extent to which behavior change interventions for weight loss maintenance in adult populations are effective [18]. Generally, per-protocol results show greater weight loss than ITT analyses. A number of large trials focusing on evidence-based approaches to weight loss maintenance have demonstrated effects on weight-related outcomes, generally not exceeding 2 kg by trial end, over time periods ranging between 6 and 12 months [42]. These include the weight loss maintenance RCT [25], DiOGenes [83], PREVIEW [84], NuLevel [85], and NoHoW [50] trials. The Look AHEAD trial produced clinically significant weight loss (≥5%) after 8 years of intensive lifestyle intervention in 50% of 2570 adults with type 2 diabetes, a patient population with a strong clinical reason for trying to achieve weight loss maintenance [86].

In this study, imputing the missing data differently tended to have an effect on absolute but not relative weight outcomes between trial arms. The more conservative our assumptions about dropout, the more favorable the resulting weight outcomes appeared; however, trial arm comparisons remained the same. When the ITT model was used with a baseline observation carried forward imputation, participants regained 40%‐50% of the weight they had lost prior to the intervention. This is consistent with similar studies in the magnitude and direction of weight regain over 12 months in response to dietary or behavior change interventions for weight management [25,83,85,86].

### Strengths and Limitations of This Study

Key strengths of this study were the 2×2 factorial design of the trial, the sample size at 3 European centers across Northern, Western, and Southern Europe, standardization and harmonization of standard operating procedures across centers, the use of minimization in randomizing participants, the duration of follow-up, the relatively low dropout rate, and the fact that weight losses prior to the intervention were verified. Limitations included the lack of a genuine baseline due to the expediency of recruiting participants who had already lost weight in the previous 12 months, the lack of balance in content type and duration between trial arms, limitations to the design of the toolkit itself that were apparent in the user acceptability and experience data, the absence of a genuine “no intervention” control, and likely contamination of the intervention itself with the content of the Fitbit app. Several of these limitations may well have impacted weight outcomes and are discussed earlier. In addition, the sample was biased toward women (1117/1627, 68.7%) and so may not be as generalizable to men who are making weight management attempts. Therefore, the small effect observed in men should be interpreted cautiously and does not alter the overall null effect in the primary ITT analysis. Finally, this trial was conducted between 2017 and 2019, with sample processing and data analysis completed in 2020. There has been a significant delay in publication due to COVID-19, organizational changes in some universities, and staff relocations among some of the key authors. However, to our knowledge, this study is still the first to use 2×2 factorial RCT in different countries to test a digital toolkit for weight loss maintenance after a period of clinically significant weight loss using self-regulation and emotion regulation; therefore, it provides a unique and timely contribution to the literature on digital interventions for weight loss maintenance, addressing a critical gap in evidence-based strategies.

### Future Directions

The NoHoW trial has demonstrated the immense benefits of using cloud-connected digital trackers to help users track their weight, physical activity, and sleep. It is notable that the salient predictor of successful weight management was engagement with the Fitbit digital weighing scales. Multicomponent behavior change interventions are, by nature, complex [87], as are physiological and behavioral responses to weight loss. It is well-established that weight loss leads to some compensation of energy expenditure and energy intake and that some behavioral changes in these components of energy balance are not always under conscious control [42]. The limited evidence from systematic reviews and meta-analyses to identify mediators of longer-term weight loss suggests that navigating from initial weight loss to weight loss maintenance requires long-term self-management of energy balance behaviors in the face of physiological resistance to weight loss [14,18-20].

The Capability, Opportunity, Motivation, Behavior model provides an overarching theoretical framework to understand the barriers and facilitators of behavior change. Specifically, the model suggests that behavior change requires capability (physiological or physical ability), motivation (reflective and automatic processes that activate or inhibit behavior), and opportunity (physical and social environment to enable behavior) [88]. Kwasnicka et al [16] have systematically reviewed theoretical explanations for the maintenance of behavior change and identified 5 overarching theoretical explanations for the maintenance of behavior change representing motives, self-regulation, psychological and physical resources, habits, and environmental or social influences on behavior. A key question is how such frameworks for reflective and automatic mechanisms of behavior change interface with the physiology of energy balance compensation in response to attempted or imposed energy deficits. Tracking compensatory changes in energy balance behaviors over time may improve the prevention of weight regain through the provision of behavioral navigation solutions, personalization of weight management interventions, and by offering a quantitative behavioral context in which psychological moderators and mediators of energy balance behaviors and body weight can be tracked [42]. Similarly, using metadata from digital interventions to track user engagement offers a powerful quantitative tool to understand the behavior of participants during the course of longer-term interventions [48]. Thus, while the NoHoW trial produced very modest results in terms of the hypothesized mechanisms of action of digital interventions, it provided unique insights into the limitations of digital intervention design and how they can be addressed and incorporated into future weight management provision.

Our understanding of factors that promote or undermine self-management of eating and physical activity is still limited but includes physiological adaptation and behavioral compensation in eating and physical activity, reactive processes related to emotions, stress, rewards, and desires that meet psychological needs. Optimizing and piloting evidence-based intervention content to the needs of individuals may improve outcomes. Objective longitudinal tracking of weight and physical activity and through mathematical modeling of energy balance components over time would provide a quantitative framework to understand the dynamics and mechanisms of action of behavior change interventions [42]. Such tracking also offers users navigational solutions. These can be combined with tracking of user engagement with intervention components to potentially improve weight management intervention design and evaluation.

### Conclusions

The NoHoW trial was a well-designed trial, adequately powered to detect changes in weight and health outcomes. In the primary ITT analysis in all participants, we found no evidence that any of the digital interventions improved weight loss maintenance compared with the active control. A small effect observed in men in exploratory subgroup analyses was not clinically meaningful and did not change the overall interpretation of the primary results.

This study is innovative in being the first large-scale, multicountry RCT to evaluate a digital-only toolkit for weight loss maintenance that integrates self-regulation, motivation, and emotion regulation within a 2×2 factorial design. NoHoW differs from prior trials by focusing on maintenance after clinically significant weight loss and by testing the independent and additive effects of digitally delivered techniques for behavioral self-regulation and emotion regulation components. The trial provides evidence on both the limitations and potential of digital behavior change interventions for long-term weight outcomes. The trial is also unique in demonstrating the potential for combined digital tracking of energy balance metrics (body weight and physical activity) and user engagement with intervention components. For health care providers, developers, and policymakers, the findings suggest that digital interventions alone may be insufficient for sustained weight management unless combined with interactive user-based feedback enabling personalization and, where feasible, human or social support. The findings emphasize the importance of routine day-to-day user experience and acceptability of digital interventions for health-related behavior change in addition to theoretical and methodological rigor.

Despite being well designed from a theoretical and evidence-based perspective, the NoHoW trial showed some limitations in consumer or user acceptability, and this study highlights some possible design aspects of the digital behavior change intervention that could be improved to enhance intervention effectiveness. These include development by an interdisciplinary team including specialists in app design and marketing or delivery; content, delivery, and structure informed by theory and evidence [51]; greater involvement from human-computer interaction experts; using optimization designs for interactive development and testing of smaller components (eg, clusters or individual behavior change techniques) to identify the combination of components that could work better together [80]; greater co-design with prospective users [89,90]; commercial partners to make digital intervention appearance and content comparable to those currently available; involvement of health care professionals; and embedding interventions and social networks to provide an ecosystem of support. These aspects of intervention design do not lend themselves well to the design and conduct of RCTs in a traditional grant-funded environment, which are constrained in time and budgetary allocation. We therefore recommend that future digital interventions for weight management are developed through academic and commercial or health care partnerships, extensively tested and refined by users and providers by the time they are evaluated through randomized trials. Novel designs, including using optimization designs and modifiable intervention content during trial designs, may also enhance effectiveness in the future.

## Supplementary material

10.2196/69634 Multimedia Appendix 1 Additional tables and figures.

10.2196/69634 Checklist 1 CONSORT-eHEALTH checklist (V 1.6.1).

## Abbreviations

CID: clinical investigation day
CONSORT: Consolidated Standards of Reporting Trials
HbA_1c_: hemoglobin A_1c_
HDL: high-density lipoprotein
ITT: intention-to-treat
NoHoW: Navigating to a Healthier Weight
RCT: randomized controlled trial
SPIRIT: Standard Protocol Items: Recommendations for Interventional Trials
