# Supplementary material for: A Digital Toolkit for Weight Loss Maintenance in European Adults (NoHoW): 2×2 Factorial Randomized Controlled Trial
Source: J Med Internet Res. 2026 May 20;28:e69634. doi: 10.2196/69634 (PMC13189573; doi:10.2196/69634)
Supplement: Multimedia Appendix 1 [file jmir-v28-e69634-s001.docx]

| **Table S1.** Sex‑stratified secondary outcomes at 12 months by intervention arm (women and men). | | | | | | | | |
| --- | --- | --- | --- | --- | --- | --- | --- | --- |
|  | **Female** | **Control** | **M** | **E** | **M+E** | **P-Motiv** | **P-Emot** | **P-MxE** |
| Body composition | Weight change (kg) | 4.53 (3.42, 5.64) | 4.75 (3.62, 5.88) | 4.47 (3.37, 5.57) | 4.73 (3.62, 5.85) | .569 | .906 | .849 |
|  | Fat free mass (kg) | 2.57 (1.46, 3.68) | 2.58 (1.52, 3.64) | 2.57 (1.74, 3.4) | 2.76 (1.71, 3.82) | .647 | .783 | .805 |
|  | Fat mass (kg) | 3.62 (2.38, 4.85) | 3.46 (2.43, 4.49) | 3.46 (2.41, 4.51) | 3.17 (2.05, 4.28) | .528 | .799 | .741 |
|  | Waist circumference (cm) | 3.33 (2.23, 4.42) | 3.2 (2.04, 4.36) | 3.27 (2.08, 4.45) | 3.4 (2.22, 4.57) | .795 | .636 | .633 |
| Biomarkers | Hba1c mmol mol | 1.31 (0.7, 1.92) | 1.23 (0.79, 1.67) | 1.55 (1.06, 2.05) | 1.48 (1.05, 1.92) | .910 | .107 | 1.000 |
|  | Hba1c % | 0.14 (0.1, 0.18) | 0.13 (0.09, 0.16) | 0.15 (0.12, 0.19) | 0.15 (0.11, 0.18) | .512 | .332 | .633 |
|  | Total cholesterol mmol/L | 0.15 (0.04, 0.26) | 0.21 (0.11, 0.32) | 0.22 (0.11, 0.34) | 0.28 (0.15, 0.4) | .468 | .202 | .808 |
|  | LDL cholesterol mmol/L | -0.11 (-0.24, 0.01) | -0.08 (-0.19, 0.02) | -0.03 (-0.13, 0.07) | -0.02 (-0.12, 0.09) | .430 | .219 | 1.000 |
|  | HDL cholesterol mmol/L | 0.16 (0.11, 0.21) | 0.15 (0.1, 0.2) | 0.16 (0.11, 0.2) | 0.17 (0.12, 0.23) | .595 | .882 | .746 |
|  | Triglycerides mmol/L | 0.27 (0.11, 0.42) | 0.25 (0.07, 0.43) | 0.25 (0.1, 0.4) | 0.21 (0.04, 0.38) | .818 | .666 | 1.000 |
|  | Systolic blood pressure mm/Hg | -0.02 (-1.83, 1.79) | -0.37 (-2.44, 1.69) | 0.19 (-1.55, 1.93) | 1.33 (-0.4, 3.05) | .789 | .119 | .096 |
|  | Diastolic blood pressure mm/Hg | 0.33 (-0.83, 1.5) | 0.13 (-1.14, 1.4) | 0 (-1.1, 1.1) | 0.69 (-0.53, 1.9) | .653 | .595 | .298 |
|  | Heart rate (bpm | 2.52 (0.95, 4.08) | 2.87 (1.36, 4.38) | 3.29 (1.96, 4.63) | 2.66 (1.19, 4.14) | .797 | .596 | .672 |
|  | Hair cortisol (log) | 0.26 (0.08, 0.44) | 0.32 (0.11, 0.54) | 0.35 (0.16, 0.54) | 0.36 (0.14, 0.57) | .700 | .486 | .820 |
|  | **Male** | **Control** | **M** | **E** | **M+E** | **P-Motiv** | **P-Emot** | **P-MxE** |
| Body composition | Weight change (kg) | 4.62 (2.97, 6.28) | 3.28 (1.6, 4.95) | 5.71 (4.06, 7.36) | 4.41 (2.73, 6.09) | .035 | .082 | .731 |
|  | Fat mass (kg) | 2.15 (0.76, 3.54) | 1.69 (0.42, 2.96) | 3.19 (1.94, 4.43) | 2.6 (1.21, 3.98) | .214 | .381 | .627 |
|  | Fat free mass (kg) | 6.6 (5.02, 8.17) | 6.35 (4.93, 7.77) | 6.67 (5.16, 8.18) | 6.61 (4.99, 8.23) | 1.000 | .813 | .736 |
|  | Waist circumference (cm) | 4.73 (3.26, 6.19) | 3.63 (2.15, 5.11) | 5.21 (3.66, 6.77) | 4.38 (2.85, 5.9) | .096 | .280 | .682 |
| Biomarkers | Hba1c mmol mol | 1.61 (1.09, 2.14) | 1.38 (0.77, 1.99) | 1.53 (0.95, 2.11) | 1.31 (0.64, 1.99) | .408 | 1.000 | .933 |
|  | Hba1c % | 0.16 (0.11, 0.21) | 0.14 (0.09, 0.19) | 0.14 (0.09, 0.18) | 0.12 (0.08, 0.17) | .311 | 1.000 | .936 |
|  | Total cholesterol mmol/L | 0.23 (0.06, 0.39) | 0.25 (0.1, 0.4) | 0.29 (0.11, 0.48) | 0.31 (0.12, 0.5) | .564 | .201 | .888 |
|  | LDL cholesterol mmol/L | 0.02 (-0.2, 0.24) | 0.06 (-0.08, 0.2) | 0.08 (-0.08, 0.25) | 0.11 (-0.03, 0.26) | 1.000 | .136 | .570 |
|  | HDL cholesterol mmol/L | 0.05 (-0.04, 0.13) | 0.06 (0, 0.12) | 0.01 (-0.05, 0.07) | 0.04 (-0.02, 0.11) | .800 | .723 | .695 |
|  | Triglycerides mmol/L | 0.33 (0.1, 0.57) | 0.26 (0.06, 0.46) | 0.34 (0.13, 0.55) | 0.25 (0.03, 0.46) | .357 | .802 | .836 |
|  | Systolic blood pressure mm/Hg | 3.97 (1.94, 5.99) | 3.26 (1.27, 5.25) | 2.98 (0.86, 5.09) | 3.77 (1.67, 5.86) | .810 | 1.000 | .738 |
|  | Diastolic blood pressure mm/Hg | 2.62 (0.88, 4.37) | 0.95 (-0.68, 2.57) | 2.22 (0.44, 4) | 1.44 (-0.12, 2.99) | .109 | .595 | .790 |
|  | Heart rate (bpm | 1.59 (-0.2, 3.38) | 1.38 (-0.36, 3.12) | 2.83 (0.68, 4.99) | 1.65 (-0.52, 3.81) | .743 | .276 | .339 |
|  | Hair cortisol (log) | 0.18 (-0.15, 0.52) | 0.16 (-0.17, 0.48) | 0.17 (-0.17, 0.51) | 0.09 (-0.26, 0.43) | .458 | .754 | .785 |

**Note.** Values are mean (95% CI) change from baseline to 12 months for waist circumference, fat mass, fat‑free mass, HbA1c, blood lipids, blood pressure, resting heart rate, and hair cortisol across intervention arms. P‑values reflect factorial main effects for the Motivation component (M), Emotion Regulation component (E), and their interaction (M×E). Population: adults enrolled in the NoHoW digital weight‑loss‑maintenance RCT across three European centres.

**Figure S1**. Longitudinal changes in body weight by trial arm (control, self-regulation and motivation, emotion regulation and self-regulation & motivation plus emotion regulation combined) for men and women in the ITT population.


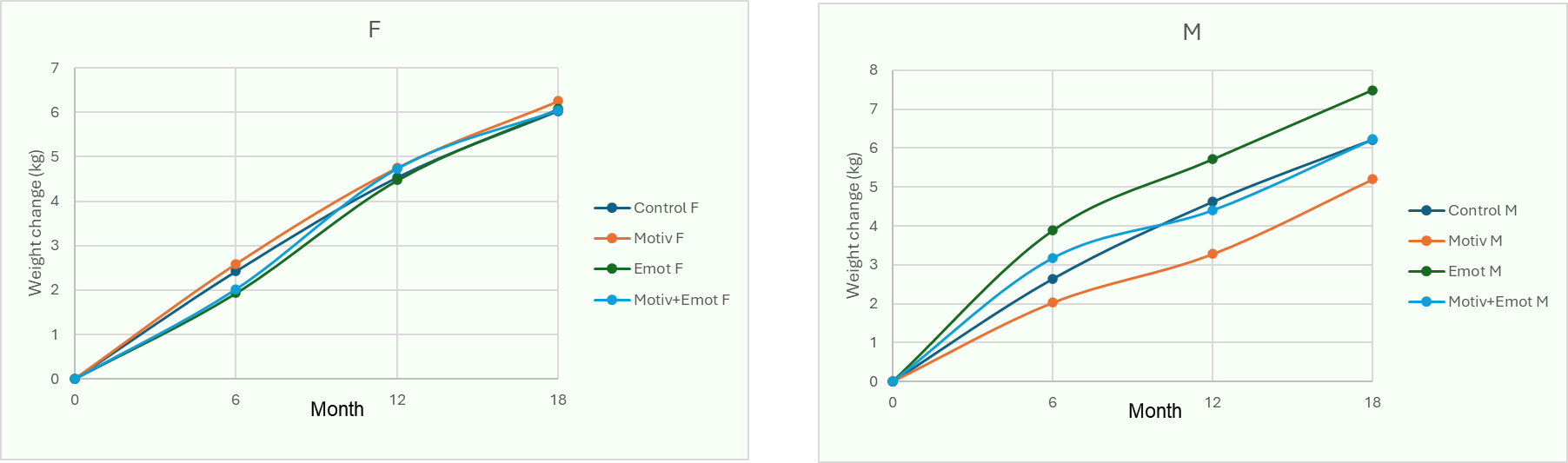


F = Female, M = Male.

**Figure S2.** Percentage of the ITT population gaining, losing or maintaining pre-trial weight loss, by trial arm (control, self-regulation and motivation, emotion regulation and self-regulation & motivation plus emotion regulation combined). Percentage weight change was calculated as (CID 3 kg – CID 1 kg) / CID 1 kg x 100. Weight category was then calculated from percentage weight change. Participants were assigned to the following weight category: >-3% weight loss (weight loser), between -2.99% and +2.99% (weight maintainer) and >+3% weight gain (weight gainer).


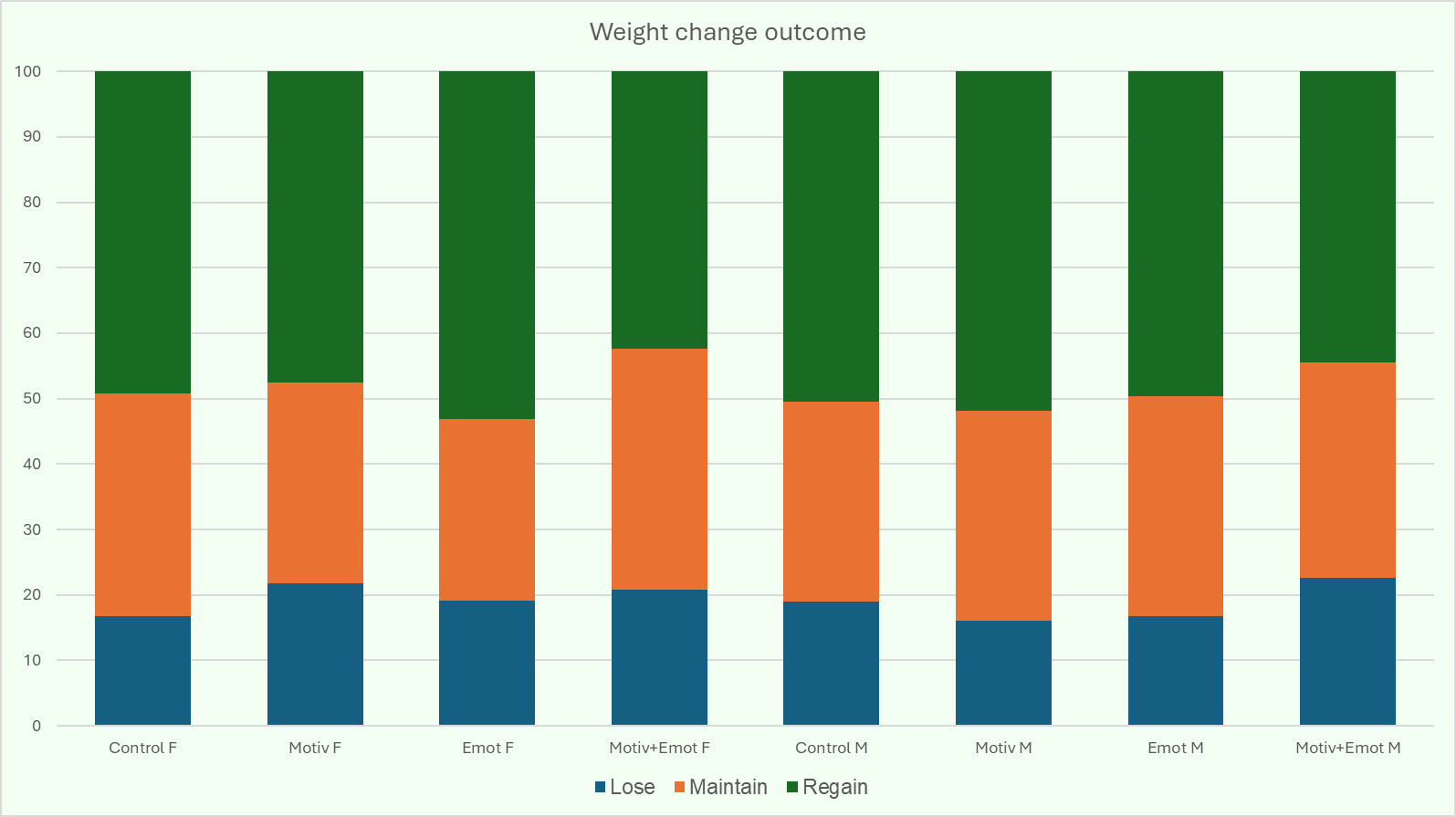


F = Female, M = Male.

| **Table S2.** Sex-stratified sensitivity analyses of 12‑month weight change (kg) for the ITT, per‑protocol, and completer populations by intervention arm. | | | | | | | |
| --- | --- | --- | --- | --- | --- | --- | --- |
| **Female** | **Control** | **M** | **E** | **M+E** | **P-M** | **P-E** | **P-MxE** |
|  |  |  |  |  |  |  |  |
| ITT | 4.53 (3.42, 5.64) | 4.75 (3.62, 5.88) | 4.47 (3.37, 5.57) | 4.73 (3.62, 5.85) | .569 | .906 | .849 |
| PP | 0.13 (-1.08, 1.34) | -0.57 (-1.98, 0.83) | 0.11 (-1.32, 1.53) | -0.46 (-2, 1.08) | .365 | .627 | .863 |
| Comp | 0.61 (-0.44, 1.65) | 0.71 (-0.36, 1.78) | 0.56 (-0.48, 1.59) | 0.64 (-0.42, 1.7) | .627 | .955 | .845 |
| **Male** | **Control** | **M** | **E** | **M+E** | **P-M** | **P-E** | **P-MxE** |
| ITT | 4.62 (2.97, 6.28) | 3.28 (1.6, 4.95) | 5.71 (4.06, 7.36) | 4.41 (2.73, 6.09) | .035 | .082 | .731 |
| PP | 0.8 (-1.01, 2.6) | -0.91 (-3.05, 1.24) | -1.23 (-3.49, 1.04) | -2.79 (-5.34, -0.24) | .043 | .029 | .930 |
| Comp | 1.09 (-0.44, 2.62) | 0.08 (-1.45, 1.61) | 1.32 (-0.22, 2.85) | 0.28 (-1.28, 1.83) | .028 | .567 | .987 |

**Note.** Values are mean (95% CI) change in body weight from baseline to 12 months for each intervention arm. P‑values reflect factorial main effects for Motivation (M), Emotion Regulation (E), and their interaction (M×E). The per‑protocol population includes participants completing ≥80% of their assigned intervention modules.

**Table S3**. Baseline measurement of the most reliable indices of physical activity and energy expenditure (Fitbit measures), and energy and nutrient intake (intake 24 measures), key scores on eating behaviour trait measures (Binge eating scale, Three factor eating questionnaire, Intuitive eating scale), well-being and quality of life (Warwick-Edinburgh wellbeing scale, EQ5D-5L).

|  |  | **Control** | **M** | **E** | **M+E** | **All** | **P-value** |
| --- | --- | --- | --- | --- | --- | --- | --- |
| **Physical activity** | IPAQ activity level | 2.81 (0.82) | 2.76 (0.81) | 2.76 (0.82) | 2.81 (0.82) | 2.79 (0.82) | .779 |
|  | Activity - self reported Moderate to Vigorous Physical Activity (min/d) | 154.61 (93.48) | 158.93 (95.01) | 169.64 (123.9) | 154.7 (88.47) | 159.41 (101.05) | .422 |
|  | Light Physical Activity (min/d) | 102.14 (35.72) | 100.3 (35.89) | 101.24 (35.21) | 101.36 (33.45) | 101.27 (35) | .967 |
|  | Moderate Physical Activity (min/d) | 101.83 (30.78) | 97.42 (35.73) | 100.83 (33.92) | 104.07 (29.58) | 101.09 (32.57) | .147 |
|  | Vigorous Physical Activity (min/d) | 105.13 (28.17) | 104.9 (29.05) | 104.54 (30.20) | 103.30 (29.74) | 104.46 (29.26) | .912 |
|  | Sitting Time (min/d) | 397.22 (228.51) | 420.34 (337.79) | 418.52 (279.23) | 419.98 (204.49) | 413.94 (267.77) | .785 |
|  | Measured FITBIT minutes sedentary (min/day) | 1104.48 (76.56) | 1106.76 (78.57) | 1110.17 (75.83) | 1103.87 (77.06) | 1106.34 (76.96) | .697 |
|  | Measured minutes light (min/d) | 277.62 (66.98) | 277.10 (63.86) | 269.94 (61.95) | 279.19 (65.72) | 275.93 (64.66) | .235 |
|  | Measured minutes fairly (min/d) | 25.09 (17.23) | 23.78 (17.57) | 24.55 (18.66) | 24.46 (17.48) | 24.47 (17.74) | .817 |
|  | Measured minutes very (min/d) | 32.81 (25.05) | 32.36 (24.81) | 35.34 (27.80) | 32.47 (24.97) | 33.26 (25.7) | .367 |
|  | Total steps/d | 10969.66 (3621.02) | 10703.94 (3601.5) | 11045.86 (3906.34) | 10905.81 (3734.63) | 10906.71 (3717.79) | .657 |
|  | Total daily energy expenditure (kcal/d) | 2763.31 (616.21) | 2775.26 (639.96) | 2779.31 (651.07) | 2782.05 (600.88) | 2775.11 (626.8) | .981 |
|  | Sleep duration (min/d) | 381.77 (74.97) | 374.72 (64.05) | 380.55 (69.75) | 381.79 (61.39) | 379.73 (67.72) | .409 |
|  | Restless duration (min/d) | 113.84 (100.79) | 119.89 (99.42) | 120.72 (101.1) | 117.41 (100.32) | 118.00 (100.35) | .776 |
| **Dietary intake** | Energy Intake (kcal/d) | 1953.55 (1093.64) | 1957.29 (873.08) | 1917.36 (913.61) | 1977.38 (941.2) | 1951.21 (958.72) | .851 |
|  | Intake fat (percentage of daily EI) | 33.24 (8.36) | 32.96 (8.29) | 33.48 (8.90) | 33.61 (9.08) | 33.33 (8.66) | .743 |
|  | Intake protein (percentage of daily EI) | 21.20 (6.14) | 20.70 (5.6) | 21.19 (5.81) | 20.79 (5.66) | 20.97 (5.81) | .508 |
|  | Intake carbohydrate (percentage of daily EI) | 40.84 (9.92) | 41.20 (10.3) | 40.76 (10.5) | 40.58 (9.83) | 40.84 (10.13) | .865 |
| **Binge Eating Scale** |  | 11.95 (7.24) | 12.51 (7.79) | 12.28 (7.55) | 11.55 (7.25) | 12.07 (7.46) | .321 |
| **Three Factor Eating Questionnaire** | Hunger | 5.71 (3.51) | 6.12 (3.42) | 5.95 (3.56) | 5.34 (3.38) | 5.78 (3.47) | .011 |
|  | Disinhibition | 8.72 (3.34) | 8.96 (3.41) | 8.98 (3.59) | 8.34 (3.31) | 8.75 (3.42) | .032 |
|  | Restraint | 10.9 (3.44) | 11.06 (3.54) | 11.2 (3.29) | 11.14 (3.47) | 11.07 (3.43) | .641 |
| **Intuitive Eating Scale** | Reliance on hunger and satiety cues | 2.82 (0.84) | 2.83 (0.9) | 2.84 (0.89) | 2.87 (0.85) | 2.84 (0.87) | .873 |
|  | Eating for Physical Reasons | 3.01 (0.58) | 3 (0.58) | 2.96 (0.60) | 2.99 (0.58) | 2.99 (0.59) | .745 |
|  | Unconditional Permission to Eat | 3.03 (0.6) | 3.04 (0.62) | 3.08 (0.63) | 3.03 (0.57) | 3.05 (0.6) | .600 |
| **Well-being and quality of life wellbeing** | | 51.20 (8.44) | 52.03 (7.94) | 51.36 (8.20) | 51.51 (7.66) | 51.52 (8.06) | .519 |
| **Overall QOL** |  | 6.31 (1.82) | 6.20 (1.46) | 6.32 (1.76) | 6.34 (1.66) | 6.30 (1.68) | .650 |
| **DASS** | Stress | 12.26 (4.27) | 12.19 (4.16) | 12.23 (4.32) | 11.8 (4.01) | 12.12 (4.19) | .374 |
|  | Anxiety | 9.02 (2.51) | 8.82 (2.34) | 9.07 (2.56) | 9.10 (2.80) | 9.00 (2.56) | .419 |
|  | Depression | 10.08 (3.95) | 9.74 (3.32) | 10.05 (3.52) | 9.94 (3.59) | 9.96 (3.6) | .544 |

Note. M = self-regulation and motivation, E = contextual behavioural aspects of emotion regulation, M+E = these factors in combination

**Table S4.** Change scores of the most reliable indices of physical activity and energy expenditure (Fitbit measures), and energy and nutrient intake (intake 24 measures), key scores on eating validated eating behaviour trait measures (Binge eating scale, Three factor eating questionnaire, Intuitive eating scale), well-being and quality of life (Warwick-Edinburgh wellbeing scale, EQ5D-5L).

|  |  | **Control** | **M** | **E** | **M+E** | **P-M** | **P-E** | **P-MxE** |
| --- | --- | --- | --- | --- | --- | --- | --- | --- |
| **Physical activity** | IPAQ activity level | 0.09 (-0.11, 0.28) | 0.09 (-0.08, 0.26) | 0.12 (-0.02, 0.26) | 0.1 (-0.09, 0.3) | .845 | .849 | 1.000 |
|  | Activity - self reported Moderate to Vigorous Physical Activity (min/d) | 38.64 (-0.5, 77.78) | 38.42 (1.04, 75.8) | 28.34 (-20.01, 76.69) | 48.2 (11.83, 84.56) | .709 | .877 | .474 |
|  | Light Physical Activity (min/d) | 11.32 (4.38, 18.27) | 14.14 (8.28, 20) | 12.14 (3.69, 20.59) | 11.76 (2.44, 21.09) | 1.000 | .574 | .558 |
|  | Moderate Physical Activity (min/d) | 10.47 (2.62, 18.32) | 11.17 (4.58, 17.76) | 6.65 (-0.23, 13.53) | 8.66 (1.97, 15.34) | .310 | .155 | 1.000 |
|  | Vigorous Physical Activity (min/d) | 6.32 (-2.14, 14.77) | 7.06 (-5.87, 19.99) | 6.3 (-6, 18.6) | 6.72 (-2.81, 16.25) | .866 | 1.000 | .899 |
|  | Sitting Time (min/d) | -34.94 (-82.87, 12.99) | -46.86 (-91.64, -2.07) | -49.54 (-93.56, -5.52) | -41.21 (-82.55, 0.13) | .749 | .927 | .429 |
|  | Measured FITBIT minutes sedentary (min/day) | 10.12 (0.49, 19.75) | 10.25 (1.38, 19.12) | 12.31 (2.25, 22.37) | 8.04 (-0.04, 16.12) | .848 | 1.000 | .619 |
|  | Measured minutes light (min/d) | -13.49 (-21.09, -5.89) | -16.39 (-22.37, -10.41) | -13.41 (-22.72, -4.1) | -13.08 (-20.24, -5.92) | .417 | .765 | .558 |
|  | Measured minutes fairly (min/d) | 1.52 (-1.44, 4.48) | 2.25 (-1.27, 5.76) | 2.4 (-0.74, 5.54) | 3.72 (-0.52, 7.97) | .389 | .519 | .986 |
|  | Measured minutes very (min/d) | -0.06 (-3.49, 3.37) | 1.75 (-2.75, 6.24) | -0.32 (-5.37, 4.72) | 0.83 (-3.22, 4.88) | .442 | .907 | .727 |
|  | Total steps/d | -519.06 (-951.04, -87.09) | -500.03 (-1071.16, 71.11) | -565.39 (-1041.3, -89.48) | -448.41 (-949.78, 52.96) | .697 | .692 | 1.000 |
|  | Total daily energy expenditure (kcal/d) | 63.80 (-3.79, 131.4) | 86.79 (17.86, 155.72) | 74.34 (10.2, 138.47) | 87.92 (12.75, 163.08) | .626 | .664 | 1.000 |
|  | Sleep duration (min/d) | -34.95 (-45.22, -24.68) | -31.13 (-40.71, -21.54) | -35.23 (-44.93, -25.54) | -34.14 (-44.65, -23.64) | .690 | .907 | .692 |
|  | Restless duration (min/d) | 65.87 (52.27, 79.47) | 66.4 (51.05, 81.75) | 66.51 (56.58, 76.44) | 64.63 (49.47, 79.78) | .676 | .715 | 1.000 |
| **Dietary intake** | Energy Intake (kcal/d) | 126.26 (-627.72, 880.24) | 135.78 (-405.8, 677.37) | -65.54 (-722.5, 591.42) | -113.61 (-786.17, 558.95) | .807 | .173 | .735 |
|  | Intake fat (percentage of daily EI) | -0.48 (-1.99, 1.03) | -0.63 (-3.27, 2.02) | -0.3 (-2.38, 1.78) | -0.78 (-3, 1.43) | .602 | .968 | 1.000 |
|  | Intake protein (percentage of daily EI) | -0.4 (-1.54, 0.74) | -1.08 (-2.25, 0.08) | -0.52 (-1.79, 0.75) | -0.7 (-1.79, 0.4) | .350 | 1.000 | .575 |
|  | Intake carbohydrate (percentage of daily EI) | 0.88 (-0.8, 2.56) | 1.81 (0.29, 3.33) | 0.85 (-0.6, 2.31) | 1 (-0.48, 2.48) | .897 | .595 | .442 |
| **Binge Eating Scale** | | 1.24 (0.41, 2.08) | 0.8 (-0.1, 1.71) | 1.01 (0.08, 1.94) | 1.1 (0.2, 1.99) | .781 | 1.000 | .411 |
| **Three Factor Eating Questionnaire** | Hunger | 0.27 (-0.27, 0.81) | 0.29 (-0.27, 0.85) | 0.31 (-0.21, 0.83) | 0.34 (-0.38, 1.07) | .710 | .886 | .850 |
|  | Disinhibition | 0.22 (-0.19, 0.64) | 0.25 (-0.12, 0.62) | 0.18 (-0.22, 0.57) | 0.24 (-0.14, 0.62) | .667 | .409 | 1.000 |
|  | Restraint | -0.34 (-0.8, 0.12) | -0.13 (-0.59, 0.33) | -0.52 (-1, -0.05) | -0.45 (-1.15, 0.26) | .977 | .120 | .737 |
| **Intuitive Eating Scale** | Reliance on hunger and satiety cues | 0.16 (0, 0.32) | 0.08 (-0.07, 0.24) | 0.17 (0.01, 0.33) | 0.14 (0.01, 0.26) | .379 | .909 | .735 |
|  | Eating for Physical Reasons | -0.1 (-0.17, -0.03) | -0.06 (-0.13, 0.02) | -0.13 (-0.21, -0.05) | -0.12 (-0.19, -0.05) | .469 | .148 | .652 |
|  | Unconditional Permission to Eat | 0.18 (0.09, 0.27) | 0.15 (0.06, 0.24) | 0.18 (0.09, 0.27) | 0.18 (0.09, 0.27) | .792 | .784 | .670 |
| **Well-being and quality of life wellbeing** | | -1.36 (-2.69, -0.04) | -1.86 (-3.59, -0.13) | -2.28 (-3.81, -0.75) | -1.71 (-3.29, -0.14) | .717 | .342 | .202 |
| **Overall QOL** |  | 0.51 (0.16, 0.85) | 0.51 (0.24, 0.79) | 0.66 (0.4, 0.93) | 0.65 (0.32, 0.97) | .680 | .185 | .783 |
| **DASS** | Stress | 0.28 (-0.56, 1.12) | 0.56 (-0.07, 1.2) | 0.59 (-0.35, 1.53) | 0.47 (-0.23, 1.18) | 1.000 | .425 | .392 |
|  | Anxiety | 0.78 (0.31, 1.26) | 0.88 (0.41, 1.34) | 0.93 (0.48, 1.39) | 0.93 (0.44, 1.42) | .602 | .267 | .843 |
|  | Depression | 0.87 (0.36, 1.38) | 1.18 (0.68, 1.68) | 1.01 (0.49, 1.53) | 0.93 (0.25, 1.62) | .552 | .712 | .471 |

**Note.** Values are mean (95% CI) change from baseline to 12 months. P‑values reflect factorial main effects for the Motivation component (M), Emotion Regulation component (E), and their interaction (M×E). Population: adults enrolled in the NoHoW digital weight‑loss‑maintenance RCT across three European centres.
